# A pre‐post intervention study: Knowledge among parents about child infections and antibiotic use facilitated by maternal and child health nurses

**DOI:** 10.1002/nop2.1330

**Published:** 2022-09-12

**Authors:** Matilde Bøgelund Hansen, Ida Scheel Rasmussen, Tina Marloth, Jens Otto Jarløv, Magnus Arpi, Dorthe Mogensen, Jette Nygaard Jensen

**Affiliations:** ^1^ Department of Clinical Microbiology Copenhagen University Hospital ‐ Herlev and Gentofte Copenhagen Denmark; ^2^ Committee for Prevention of Hospital Infections Capital Region of Denmark Copenhagen Denmark

**Keywords:** antibiotic, health literacy, health visitor, infant, information booklet, maternal and child health nurse

## Abstract

**Aims:**

To investigate parent's knowledge and beliefs of common infections and antibiotics in children before and after an educational intervention provided by maternal and child health nurses. Second, to investigate sociodemographic differences in parent's knowledge before and following the intervention.

**Design:**

A prospective pre‐post intervention study. The intervention consisted of a booklet with information about childhood infections delivered by maternal and child health nurses.

**Methods:**

The study population included 344 parents with a child born during 2017 and residing in three Danish municipalities. Knowledge about infections and antibiotics were collected quantitatively through an online questionnaire before and after the intervention (August 2017–November 2018) and analysed using linear mixed models.

**Results:**

Parental knowledge increased after the intervention. Parents with lower education and born in Denmark compared to parents with higher education and born in other countries experienced a higher increase in knowledge from baseline to follow‐up.

## 1 INTRODUCTION

Antibiotic resistance in bacteria is judged to be one of the biggest threats to the public health globally (Ventola, 2015; World Health Organization, 2017). Unnecessary use of antibiotics has a major responsibility for the increasing development of antibiotic resistance that results in increased mortality and morbidity for the patients (Goossens et al., 2005; World Health Organisation, 2012).

Despite a declining use of antibiotics in young children in Denmark, Danish children are prescribed more antibiotics than children in the other northern countries: Sweden and Norway (DANMAP, 2020; Hansen et al., 2021; NORM/NORM‐VET, 2020; Swedres Svarm, 2020). This difference cannot be explained by variations in incidences of common acute infections and/or healthcare systems (Mossialos & Wenzl, 2016). Inappropriate use of antibiotics among children may cause serious adverse short‐term and long‐term side effects (Ramirez et al., 2020). Recently, more studies have focused on the long‐term effects of antibiotic use in early life. Use of antibiotics during infancy has been suspected to increase the risk of obesity, neurocognitive outcomes and asthma among other illness developed later in life (Ramirez et al., 2020; Slykerman et al., 2017). Hence, it is crucial to eliminate inappropriate use of antibiotics particularly among infants and young children.

### 1.1 Background

Studies have examined parents communication with GPs and how this contributes to overuse of antibiotics (Stivers, 2021). Stivers (2021) found that parents use communication strategies that, although not necessarily intentional, are consistently understood by GP's as a pressure to prescribe antibiotics. For example, parents may question a GP's recommendation for nonantibiotic treatment, which may lead to inappropriate prescribing of antibiotics. This suggest that improving parents knowledge and beliefs of appropriate antibiotic use can modify this perceived demand for antibiotics from parents and subsequently reduce the amount of inappropriate antibiotics prescribed for young children. Educational efforts or interventions that address information about infections and antibiotics to parents with young children have been proposed as part of a solution (Trepka et al., 2001; Vaz et al., 2015) and proved to be effective in limiting the inappropriate use of antibiotics (Andrews et al., 2012; Strama Halland, 2010). However, to our knowledge, no studies have used maternal and child health nurses (MCHNs) as knowledge facilitators to implement information about common childhood infections and antibiotic use to new parents (i.e. parents with a child aged <1 years). Therefore, this study intended to tailor and implement an intervention in a Danish context to increase parental knowledge about child infections and antibiotic use by developing an informational booklet and using MCHNs as knowledge facilitators.

The overall objective of this study was to evaluate knowledge and beliefs regarding common infections and antibiotics among new parents before and after exposure to an educational intervention facilitated by MCHNs. Secondary objectives were to assess sociodemographic differences in knowledge and beliefs at baseline as well as in change in knowledge and beliefs from baseline to follow‐up.

## 2 THE STUDY

### 2.1 Design

This study was designed as a prospective pre‐post intervention study using quantitative data.

**FIGURE 1:**
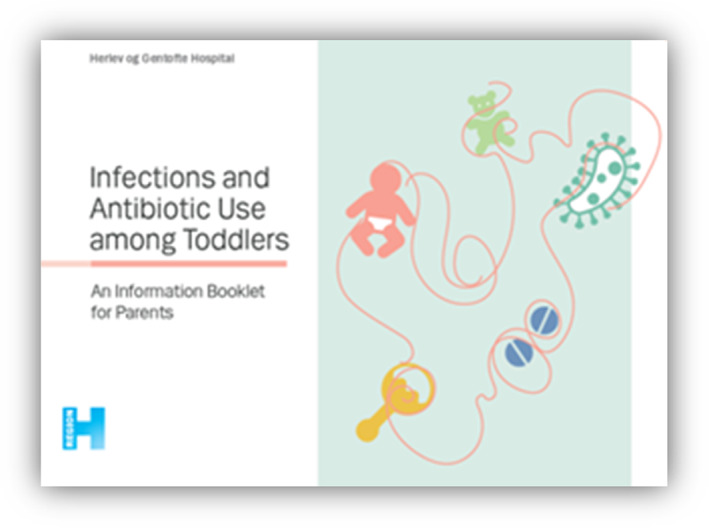
Front page of booklet to inform new parents about common childhood infections and treatment options

The intervention consisted of a booklet written in Danish or English for new parents (Antibiotic Stewardship, & Implementaion Research Unit, Department of Clinical Microbiology, Herlev and Gentofte, 2017). The booklet was developed by four MCHNs, a clinical microbiologist, an infection control nurse and a GP. The content included evidence‐based information on symptoms and management of the most common infections among children that was based on recommendations from the national Health Authorities and review of international literature (Danish Health Authority, 2020). Importantly, this information was conveyed in a language that was easy to understand. The design of the booklet included inviting colours and a material that was robust and easy to wipe off (Figure 1). The purpose of the booklet was to make the parents more informed about infections and antibiotics before and when consulting the GP. The booklet included a section stating that the GP is the expert when diagnosing the child and prescribing the treatment, however, also underlined that the parent's knowledge was important when having a dialogue with the GP. Once the MCHN came to visit the parents' home at 8 months post birth, the MCHN introduced the booklet to the parent.

Before the initiation of the intervention, a half‐day educational seminar was held to all MCHNs (*N* = 40) working in the included three municipalities. The booklet was introduced at the seminar along with the scientific evidence behind the content of the booklet. Besides the seminar, all MCHNs received a compendium with extended evidence‐based material related to the content in the booklet. The goal of the educational seminar was to prepare the MCHNs as ambassadors of the intervention.

### 2.2 Participants

The study was carried out in three municipalities in the Capital Region of Denmark. The municipalities were selected at random within the Capital Region of Denmark. Parents of children born during 2017 and residing in one of the three municipalities were the target population in this study. All families in Denmark are offered a visit by a MCHN free of charge at 1 week, 1 month, 2 months, 4–6 months and 8–10 months post birth. MCHNs hold an education as a registered nurse (3.5 years) as well as an extensional education as a MCHN (1.5 years). They are employed in the municipality and their work covers prevention of diseases and health promotion among children. Parents were invited to participate through the local MCHNs during one of the first visits post birth by signing a formula and giving consent to participate in this study. Preferred language (Danish/English) and contact information were collected.

### 2.3 Data collection

An electronic self‐administered baseline questionnaire and follow‐up questionnaire were developed in Danish and English. The questionnaire was designed in the survey software Enalyzer and sent by email to the parents. The baseline survey was sent to the parents before the child was 6 months old, and the follow‐up survey was sent to the same parents at child age 12–13 months. The data collection took place from August 2017–November 2018. Two reminders were sent to non‐responders.

The baseline survey was structured into different sections. One section of the questionnaire covered the parent's demographic data such as the parent's age, country of birth, highest attained education and number of children living at home. Another section covered the parent's knowledge and beliefs (hereafter referred to as knowledge) of common infections and antibiotic use comprised by 22 questions with response options ‘Agree’, ‘Do not agree’ and ‘Do not know’. The follow‐up survey included questions on whether the parent had received and read the booklet along with the 22 knowledge questions from the baseline survey. Twenty‐one out of the 22 questions on knowledge about infections and antibiotics were included in this study (Table 2). The question ‘Mistaken use of antibiotics can increase the resistance of humans to them’ was removed since it intended to measure the same content as the question ‘Humans can be resistant to antibiotics’.

### 2.4 Ethical considerations

According to Danish legislation, no ethical approval is needed for this study because it seeks to evaluate the implementation of knowledge with existing consensus and because it uses survey‐based data (National Research Ethics Committees, 2013). Before responding to the survey, parents were informed about the purpose of the study, that participation was voluntary, and that they could withdraw from the study at any time. They were also assured of the confidentiality of the data to be collected, and that personal information that could be used to identify them would be changed before results were published.

### 2.5 Data analyses

Data were analyzed using IBM SPSS Statistics for Windows, Version 25.0 (IBM Corp, 2017). We assessed the difference in number of correct answers to single items of the knowledge questionnaire at baseline and follow‐up. Besides, an overall infection and antibiotic knowledge score were calculated for each parent. This score was the total number of correct answers (0–21). Parents that responded to at least 17 of 21 knowledge questions were included. ‘Do not know’ answers were categorized as incorrect for all analyses. Linear mixed‐model regression analysis was used to assess difference between baseline and follow‐up in the overall infection and antibiotic knowledge score (Gueorguieva & Krystal, 2004). We adjusted for number of question answered to take account of the difference in number of question answered between baseline and follow‐up. Linear mixed model is an extension of standard linear regression that allows to take account of correlation within repeated measures by specifying that residual errors are correlated. Also, the linear mixed model do not require that each subject have available data at both timepoints and therefor take advantage of all available data. Linear mixed model regression analysis was also performed to investigate sociodemographic differences in baseline knowledge as well as in change in knowledge from baseline to follow‐up. Five sociodemographic variables (age, education, medical/healthcare‐related education, number of children and country of birth) were analysed in five separate models. An interaction term between the sociodemographic variable and time was included to assess sociodemographic differences in changes in knowledge before and after the intervention. We adjusted for number of questions answered and relevant confounders which were considered to influence the sociodemographic variable of interest and knowledge of infection and antibiotics.

### 2.6 Validity and reliability

The questions from the survey were based on previous surveys and face validated by experts in clinical microbiology (André et al., 2010; World Health Organization, 2015). Twelve healthcare workers pilot tested the survey. Cronbach's alpha was calculated to perform a reliability analysis of measured knowledge and beliefs. The Cronbach's alpha value was 0.83, underlining a high level of internal consistency.

## 3 RESULTS

### 3.1 Description of the study participants

In total, 348 (73%) of 474 parents responded to the baseline survey of which 344 answered enough knowledge statements. A total of 241 of baseline participants responded to the follow‐up survey resulting in a loss to follow‐up at rate 30% from baseline to follow‐up. At follow‐up, three parents were excluded due to missing information on knowledge statements and 95 were excluded as they replied that they had not received the booklet. Therefore, the total number of observations was 344 at baseline and 143 at follow‐up.

One hundred and twenty‐four of the 143 parents that were included at follow‐up reported that they had read in the booklet. The parents were predominantly women (95%) and the mean parent age was 34 years (Table 1). The majority of the parents had more than one child (64%) and were living with a partner (97%). A high proportion of the parents had a higher education (82%) and more than one‐fifth of the parents were educated in the medical field. The mean age of the children of the participating parents were 5 months at baseline and 12 months at follow‐up.

**TABLE 1:** Description of study population

Variables	Baseline		Follow‐up	
	Number	%	Number	%
Total	344		143	
Parent characteristics				
Gender				
Female	325	94.5	138	96.5
Male	19	5.5	5	3.5
Missing	0		0	
Age				
22–30 years	86	25.0	30	21.0
31–40 years	228	66.3	105	73.4
>41 years	30	8.7	8	5.6
Missing	0		0	
Education				
Primary, secondary or vocational	<65	<20	21	14.8
Highest attained education	279	81.6	121	85.2
Missing	<5	<1.5	1	
Medical or healthcare‐related education				
Yes	80	24.3	30	21.7
No	249	75.7	108	78.3
Missing	15		5	
Number of children				
1	125	36.3	55	62.5
>1	219	63.7	88	38.5
Missing	0		0	
Single parent				
No	332	96.5	136	95.1
Yes	12	3.5	7	4.9
Missing	0		0	
Parental country of Birth				
Denmark	299	86.9	124	86.7
Other country	45	13.1	19	13.3
Missing	0		0	

### 3.2 Parental knowledge regarding common infections and antibiotic use

The distribution of correct answers with regard to individual knowledge questions is presented in Table 2. The number of correct responses was higher at follow‐up compared with baseline for 20 of the 21 questions. At baseline, the average number of questions that were correctly answered among parents were 13.46 of 21 questions. The average number of questions with correct answer for parents increased significantly with 1.35 (95% CI: 0.91; 1.79) questions with correct answer to an average of 14.81 correct answers at follow‐up (results not shown).

**TABLE 2:** Distribution of parent's correct responses to questions on infections and antibiotics before and after the educational intervention by MCHNs

Knowledge question	% correct answers		Difference (percentage points)
	Baseline, *N* = 344	Follow‐up, *N* = 143	
Antibiotics are effective against the bacteria that may cause illness	81	89	8
Antibiotics are effective against the viruses that may cause illness	66	80	14
Colds are caused by bacteria	67	80	13
Colds are caused by virus	82	87	5
Antibiotics speed up recovery from a cold	82	85	3
If the nasal catarrh (snot) from a head cold is coloured (e.g. greenish/yellowish), you often need antibiotics to get rid of the cold, as the colour indicates bacteria	42	49	7
It is always appropriate to use antibiotics when your child has tonsillitis otherwise he/she might catch something more serious	56	66	10
Inflammation of the ear in children younger than 6 months should almost always be treated with antibiotics	19	18	−1
Inflammation of the ear in children younger than 2 years should almost always be treated with antibiotics	27	40	13
Antibiotics can kill both the beneficial bacteria and the non‐beneficial bacteria in the body	68	79	11
Skin reaction (e.g. rash) can be a side effect of antibiotics	57	63	6
Antibiotics can cause imbalance in the body's own bacterial flora	73	83	10
If my child feels better after half of the treatment with antibiotics I usually interrupt the treatment	79	85	6
Unnecessary use of antibiotics can reduce the body's own capacity to fight off infections	77	89	12
Humans can be resistant to antibiotics	29	46	17
Bacteria can be resistant to antibiotics	89	91	2
Mistaken use of antibiotics can increase the resistance of bacteria to them	82	88	6
The use of antibiotics among animals can reduce the effect of antibiotics among humans	48	57	9
Bacteria that are resistant to antibiotics can be spread from person to person	62	67	5
Antibiotic resistance is an issue that could affect me or my family	75	79	4
In the end, antibiotic resistance could lead to some infections being incurable	82	90	8

Parental characteristics according to baseline knowledge as well as change in knowledge from baseline to follow‐up are presented in Table 3. Having a higher age, higher educational level, a medical/healthcare‐related education or more than one child was associated with higher parental knowledge at baseline than parents with lower age, lower educational level, without an education related to health care or only one child. Level of education and country of birth were the only parental characteristics that were significantly associated with changes in knowledge from baseline to follow‐up. Parents with a lower educational level or parents born in Denmark experienced a greater increase in knowledge from baseline to follow‐up than parents with higher educational level or parents born in other countries, respectively.

**TABLE 3:** Results from linear mixed model regression assessing parent's sociodemographic characteristics and their baseline knowledge score and change of knowledge score from baseline to follow‐up

	Baseline			Follow‐up		
	Score	95% CI	*p*	Score	95% CI	*p*
Age						
22–30 years	Ref		<.001	Ref		.310
31–40 years	2.343	0.627; 4.060		0.163	−1.903; 2.228	
>41 years	2.608	1.583; 3.632		−0.742	−1.847; 0.363	
Education^a^						
Highest attained education	Ref		.043	Ref		.046
Primary, secondary or vocational	−1.791	−2.940; −0.641		1.246	0.024; 2.468	
Medical or healthcare‐related education^b^						
No	Ref		<.001	Ref		.937
Yes	2.475	1.481; 3.469		0.0431	−1.034; 1.121	
Number of children^c^						
1	Ref		.001	Ref		.810
>1	1.506	0.587; 2.425		−0.112	−1.031; 0.807	
Country of birth						
Other country	Ref		.257	Ref		.003
Denmark	−0.234	−1.575; 1.106		1.942	0.686; 3.197	

^a^ Adjusted for parent age and country of birth.

^b^ Adjusted for parent age, country of birth and education.

^c^ Adjusted for parent age, country of birth and medical/healthcare‐related education.

## 4 DISCUSSION

The main finding of this study was that overall knowledge regarding common infections and antibiotic use among new parents increased after an educational intervention facilitated by MCHNs. Parents with lower age, lower educational level, without a medical or healthcare‐related education or only one child had less overall knowledge at baseline than parents with higher age, higher education level, a medical/healthcare‐related education or more than one child, respectively. Parents with lower educational level or parents born in Denmark experienced a higher increase in knowledge from baseline to follow‐up than parents with higher educational level or parents born in other countries.

### 4.1 Findings in relation to the existing literature

The results of this study are in line with a review by Neill et al. (2015), that found an increase in parental knowledge after an educational intervention in eight of nine studies (Neill et al., 2015). The review found that interventions consisting of written together with verbal information were more successful in retaining parental knowledge, hypothesizing that provision of verbal information may signal healthcare professionals' endorsement of the information. Other factors that have been highlighted for successful interventions relates to the content (focussed, information on multiple childhood symptoms), timing (before child illness) and format (multifaceted and cartoon illustrations) of the intervention (Andrews et al., 2012; Neill et al., 2015).

Parental knowledge of common infections and antibiotics seemed already relatively high at baseline in this population. In the general population in Denmark, it is found that 51% believe that antibiotics kill viruses while this study showed that only 33% of new parents seemed to believe that antibiotics are effective against viruses (European Commission et al., 2018). Likewise, differences in knowledge according to parental education, age and number of children have previously been identified (Bosley et al., 2018). For example, a Danish study by Jensen et al. found that the higher the education of the parents, the lower the risk of high antibiotic consumption among children (Jensen et al., 2016). Parents with higher education might have more resources to access information on proper use of antibiotics. Opposite, younger parents and parents with fewer children often have less experience and confidence in caring for an ill child. This underlines the importance of healthcare professionals to make information and knowledge readily accessible by use of simple terms and adequate explanations as well as allowing parents the opportunity to ask questions. Increased knowledge about the nature of common infections and confidence in coping with infections in children may reassure new parents and impact expectations and perceptions of common childhood infections which could influence parental behaviour.

Our findings indicated that parents born in Denmark experienced a greater gain in knowledge compared to parents born outside Denmark following the intervention. The reasons for this difference are unknown. One explanation may be that different traditions and beliefs around infections and antibiotics across geographical locations interact with the rationale in the booklet. Other explanations may relate to the situations around the introduction of the booklet or the methods applied.

### 4.2 Limitations

Several limitations of this study should be considered. First, the prospective pre‐test post‐test intervention study design makes it vulnerable to bias and the impact of the intervention should be interpreted with caution. It is possible that other initiatives or information may have increased the level of knowledge of parents, but to our knowledge, there were no other local or national initiatives targeted parents understanding of infections and antibiotic in the study period.

Another limitation of this study is the small study population which increased the statistical uncertainty. However, the mixed model regression analysis include all available observations and a post hoc analysis showed no difference in dropout from baseline to follow‐up in relation to participants sociodemographic background or knowledge score at baseline.

Furthermore, it is highly likely that our results are prone to selection bias. Parents with an interest in the topic of infections and antibiotics might be more likely to receive the booklet and at the same time parents with interest in the topic might be more likely to have higher level of knowledge. The socio‐economic statuses of the participating municipalities are ranked among the most wealthy municipalities in the Capital Region, indicating that the inhabitants are generally well educated, have a high income and that a high proportion of the population of working age are at the labour market (Juel Lau et al., 2018). This might explain the finding of a higher knowledge than in other populations with more diverse socio‐economic backgrounds (Svendsen et al., 2020). Therefore, the potential to increase knowledge and influence new parent's behaviour might have been higher in other municipals with lower knowledge levels as a starting point.

Finally, bias might also result from misclassification since data in this study was based on self‐reported questionnaires. The surveys were developed in Danish and English and hence parents that speak and read English well could participate on equal terms as Danes. Anyhow, it is possible that reporting of exposure to the booklet and knowledge level is misclassified among parents, that do not speak or read Danish or English if they did respond to the survey. This limitation might contribute to the finding that parents born outside Denmark experienced a smaller increase in knowledge than parents born in Denmark.

## 5 CONCLUSION

This study indicates that it may be possible to improve knowledge about common childhood infections and antibiotic use among new parents with an educational intervention facilitated by MCHNs and supported by experts in infection prevention and microbiology. The potential of using MCHNs to inform new parents about common infections and antibiotics are possibly undervalued. MCHNs in Denmark visit all new parents repeatedly and may therefore be able to establish a solid and trustful basis for informing parents about infections and antibiotics among infants and toddlers. Eventually, parents may use this knowledge in the decision to consult the GP and/or when discussion treatment options with the GP. This study does not provide knowledge on how the intervention influenced parental behaviour. Future studies should explore in a more socio‐economic diverse population how, why and under which circumstances interventions affect parents' abilities to assess and manage common childhood infections. Future studies should also explore how culture may interact with interventions that address parents' beliefs about and mangement of common childhood infections to more effectively target parents from different cultures in future interventions.

## AUTHOR CONTRIBUTIONS

JNJ, ISR, TM, JOJ, MA and DM contributed to the conceptualization and design of the study. JNJ, ISR and MBH were responsible for the data collection and analysis. JNJ, ISR and MBH wrote the first draft of the manuscript. TM, JOJ, MA and DM criticially reviewed the manuscript. All authors have read and approved the final manuscript.

All authors have agreed on the final version and meet at least one of the following criteria [recommended bythe ICMJE (http://www.icmje.org/recommendations/)]:

• substantial contributions to conception and design, acquisition of data or analysis and interpretation of data;

• drafting the article or revising it critically for important intellectual content.

## FUNDING INFORMATION

This work was supported by ‘Tværspuljen’ under Grant number P‐2016‐2‐03 and the Danish Ministry of Health under Grant number 1608956.

## CONFLICT OF INTEREST

No conflict of interest has been declared by the authors.

## ETHICAL APPROVAL

According to Danish legislation, no ethical approval is needed for this study because it seeks to evaluate the implementation of knowledge with existing consensus and because it uses survey‐based data (National Research Ethics Committees, 2013)

## Data Availability

Research data are not shared.
